# Scoring shoulder ulcers in breeding sows – is a distinction between substantial and insubstantial animal welfare-related lesions possible on clinical examination?

**DOI:** 10.1186/s40813-018-0108-3

**Published:** 2019-01-22

**Authors:** Daniel Meyer, Marion Hewicker-Trautwein, Maria Hartmann, Lothar Kreienbrock, Elisabeth grosse Beilage

**Affiliations:** 1 Field Station for Epidemiology in Bakum, University of Veterinary Medicine Hanover, Foundation Buescheler Str. 9, 49456 Bakum, Germany; 2 Department of Pathology, University of Veterinary Medicine Hanover, Foundation Buenteweg 17, 30559 Hanover, Germany; 3 Institute for Biometry, Epidemiology and Information Processing (IBEI), University of Veterinary Medicine Hanover, Foundation Buenteweg 2, 30559 Hanover, Germany

**Keywords:** Clinical score, Histopathological score, Necrosis, Neuroma, Proliferation of wound margins, Scab, Scar tissue, Skin layer

## Abstract

**Background:**

Shoulder ulcers in breeding sows that are restricted to the superficial skin layers, epidermis and dermis are usually classified as insubstantial animal welfare-related lesions. These less-severe lesions need to be differentiated from more-severe wounds that also involve the subcutis and the underlying bone, commonly evaluated as substantial animal welfare-related lesions. Scoring schemes based on clinical or histopathological findings are available, but the consistency between both types of findings has not been definitively evaluated. The present study was designed to compare clinical findings for various stages of shoulder ulcers with accompanying histopathological evaluation. A validated histopathologic score (Score-H) classifying the tissues involved in the different stages of shoulder ulcers was set as the reference standard.

**Results:**

Testing the histopathological scores for associations with various clinical findings resulted in a clinical score (Score-C) that could be segregated into four stages. Stage I is characterised by intact skin without any ulcerative lesions. Stage II, representing ulcerative lesions restricted to the superficial skin layers, can be predicted with a probability of 90% when a scab with diameter less than 1.2 cm is present. Stage III, representing ulcers involving the entire skin and sometimes the underlying bone, can be identified by the diameter of the scab (DOS) and/or proliferation of wound margins (powm) and/or increase of tissue volume (mass). To achieve a probability of 90%, the DOS needs to be 8.3 cm when mass and powm are absent. DOS, when accompanied by powm and mass, needs to be only a minimum of 1.9 cm for a correct classification with a 90% probability. Stage IV represents skin without open wounds but with scar tissue indicative of a former shoulder ulcer.

**Conclusions:**

Based on the association with the histopathological findings as the reference standard a clinical score (Score-C) for the categorisation of shoulder ulcers in sows was developed. This score enables veterinarians and farmers to discriminate shoulder ulcers restricted to the superficial skin layers from ulcers involving all skin layers and sometimes even the underlying bone, which must be assessed as substantial animal welfare-related lesions.

**Electronic supplementary material:**

The online version of this article (10.1186/s40813-018-0108-3) contains supplementary material, which is available to authorized users.

## Background

### Definition and description

The term ulcer refers to the loss of the epidermis and basement membrane with exposure of the dermis [1]. Shoulder ulcers or decubital sores are lesions located over the tuber spina scapulae frequently seen in sows housed in intensive production systems. The lesion is comparable to decubital lesions in humans [2]. Shoulder lesions in sows can be restricted to the superficial to deeper layers of the dermis or involve the underlying bone tissue. Lesions affecting the deeper layers or the bone tissue are suggested to be associated with acute pain caused by an inflammatory reaction [3], as well as by traumatic neuromas that may be present even after initial wound healing [4].

The herd prevalence reported in studies from several Scandinavian countries and North America varied from 8 to 34% [5–8]. The main risk factors for the development of shoulder ulcers in sows include lameness and a low Body Condition Score (BCS) [7–9]. Scar tissue, a sign of a previous shoulder ulcer, increases the probability of future findings [5, 7–10].

### Pain and welfare

The National Ulcer Advisory Panel (NUAP) has determined that humans usually feel pain from decubital lesions [11]. People suffering from pressure sores describe the pain on a scale ranging from “discomforting” to “distressing” and up to “horrible” [1]. The feeling of pain is most likely induced by the inflammatory reaction, as well as by traumatic neuromas that appear to be responsible for so-called “phantom pain”. As the nociceptive systems of human beings and pigs show similar traits [12], and as inflammatory reactions and traumatic neuromas are found in the histological examination of shoulder ulcers [4], it has been suggested that shoulder ulcers are painful for sows as well [10]. The development of pain related to the detection of traumatic neuromas has been extensively described in pigs after tail docking [4, 13, 14].

The International Association for the Study of Pain defines pain as “an unpleasant sensory and emotional experience associated with actual or potential tissue damage, or described in terms of such damage” [15]. Animals adapt their behaviour in order to avoid damage [16]. Behavioural adaptions are important in terms of pain analysis. In sows with shoulder lesions, increased rubbing behaviour suggested to be associated with pain has been observed after palpation of the affected shoulders [17]. Correlation between pain-associated reactions after palpation of the shoulders and the depth of the lesion was also determined [18].

### Pathogenesis

Shoulder ulcers in sows develop nearly exclusively at the time surrounding farrowing [19]. Pressure, shear forces, friction, and the duration of lying periods surrounding farrowing are essential factors for the initiation of the process of tissue damage [2, 20, 21]. The pathogenesis is generally described as a compression of blood vessels in the tissue overlying the tuber spina scapulae, which leads to ischaemia. The occlusion of blood and lymph vessels is followed by an aggregation of products of catabolism. This results in local cell necrosis, with inflammation and proliferation that can include all tissue layers [2]. The progression of shoulder ulcers in sows is considered a process stemming from the epidermal layer to bone tissue (“top to bottom”) [22, 23].

### Histopathological scoring (score-H) of shoulder ulcers

The histopathological scoring (Table 1) allows the differentiation of the affected skin layers [23]. For a more compact overview, the table has been modified slightly.

**Table 1:** Criteria for the histopathological characterisation of shoulder ulcers (modification of Jensen 2009)

	Stage					
Tissue	0	1	2	3	4	5^a^
Epidermis	Intact	Necrotic or missing	Necrotic or missing	Necrotic or missing	Necrotic or missing	Often absent
Dermis		Necrosis limited to the superficial dermis	Necrosis of the major part, residues of hair follicles and glandular structures	Necrotic or missing		Granulation tissue, fibroplasia
Subcutis		No lesions	No necrosis	Necrotic, excessive granulation tissue, microabscesses		
Bone			Slight proliferation of cambium possible	Reactive cambium and thickened, periosteal bone formation	Demarcated by excessive granulation tissue, “unprotected”^b^	Often thickened, sometimes granulation tissue
Muscle^c^			No lesion	Suprascapular muscles with fibrosis	Severely atrophied muscles	No lesion

^a^Healed ulcer

^b^Overlying tissue is missing

^c^Parts of surrounding shoulder girdle muscles (*M. trapezius* or *M. deltoideus*)

### Clinical scoring of shoulder ulcers

Various schemes for scoring shoulder ulcers in sows by clinical examination have been published (Table 2). The scoring systems vary in the number of stages, and the precision of the clinical parameters are evaluated. Several are simply related to the diameter of a lesion or the amount of scab formation. Others additionally include signs of inflammation or repair of the skin, such as reddening or fibrosis.

**Table 2:** Clinical scoring systems for the classification of shoulder ulcers in sows

Stage	Lund 2006^a^	Zurbrigg 2006	Baustad, Frederiksen 2006	Lund 2003, Jensen 2009^b^	Bonde 2004	Welfare Quality® 2009	Stage	Grading system in Denmark^c^
0	No skin lesion	No lesion/scarring	Normal skin or fresh fighting wounds	Intact skin, no ulceration	–	–	0	No lesion or lesion < 2 cm
1	Lesion limited to epidermis, moderate scab	No current lesion but previous scarring	Ulcer with skin merely affected	Ulcer limited to epidermis (necrotic or sloughed), covered with a scab	No lesion	No lesion	1	Small lesion 2–5 cm in diameter
2	Lesion involves dermis; extensive scab; little fibrosis/granulation	Skin reddening	Ulcer penetrating the skin	Ulcer including dermis; sometimes scab; bordering granulation tissue/fibrosis	Scratches	Old lesion, healing wound, reddening		
3	Lesion penetrates to subcutis; intense granulation	Broken skin < 2.3 cm	Ulcer extending to subcutis; granulation tissue possible	Ulcer including subcutis, sometimes scab; heavy formation of fibrosis/granulation tissue	Wounds	Lesion/open wound	2	Lesion > 5 cm; Thickened wound perimeter
4	Ulcer extending to bone; periosteal bony proliferation	Broken skin ≥2.3 cm	Ulcers with exposure of the scapula	Ulcer with exposed bone, proliferation of osseous tissue	–	–		

^a^Modified by Jensen and Svendsen 2006

^b^Cited by Herskin 2011

^c^Nielsen personal correspondence

The present study was designed to develop an on-farm usable clinical score (Score-C) for shoulder ulcers in sows by comparing clinical symptoms with the according histopathological findings assessed by a validated histopathologic score (Score-H)

## Material and methods

The study was conducted from April 2015 to February 2016 in North-Western Germany. At a slaughterhouse, sows with and without signs of shoulder ulcers were clinically examined, and corresponding shoulder tissue samples were collected post-mortem.

### Clinical examination

**Fig. 1:**
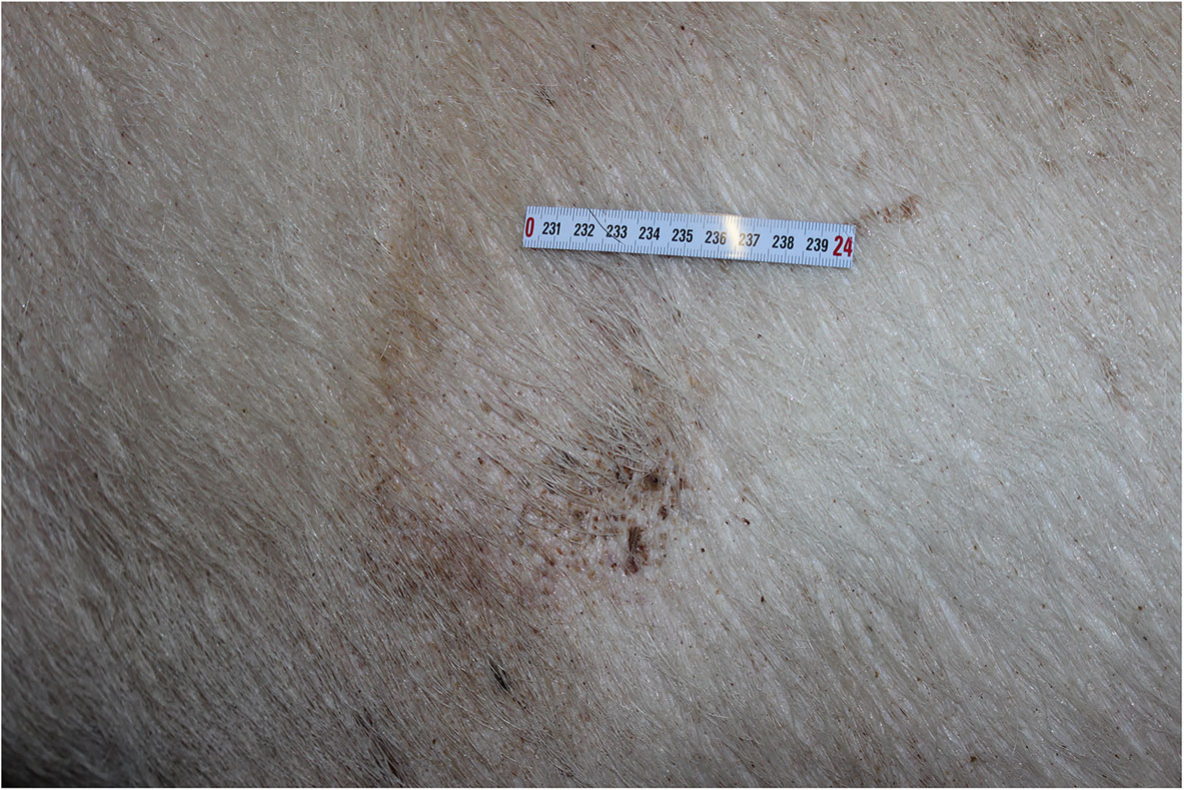
Top view of the left shoulder of a sow with an ulcer stage 5 (scar tissue)

In the waiting area of the slaughterhouse, the shoulders of the sows were clinically examined, and the diameter of the scab (DOS) was measured with the help of a measuring tape. All examinations were done by the first author. The findings were recorded using a standardised scheme (Table 3) and include photo-documentation (Fig. 1). All sows were individually marked by ear tags.

**Table 3:** Potential clinical findings in sow shoulders

Skin	Hairless		Yes/No
	Intact		Yes/No
	Reddening		Yes/No
Skin lesion	Scratch		Yes/No
	Scratches (≥ 2)		Yes/No
	Scar		Yes/No
	Proliferation of wound margins (powm)		Yes/No
	Scab		yes/no
		Diameter (cm)	
	Mass	> 4 cm	Yes/No
		Fluctuation	Yes/No
		Relocatable	Yes/No

### Collection and processing of shoulder tissue samples

Carcasses from animals with the specific ear tags that had been selected at the live animal arrival platform were separated, and a 20–30 cm × 30–40 cm piece of the shoulder (including the top of the spina scapula as well as the soft tissue layers) was removed with the help of a circular saw. The tissue was cut to a size that did not exceed 20 cm in width or length and stored in 10% neutral buffered formalin.

### Histopathological examination

From each of the formalin-fixed tissue pieces, three smaller tissue samples were selected for histology. Two soft tissue samples measuring 1.5–2.5 cm × 2–3 cm were taken from the transition from the macroscopically unaffected tissue to altered tissue. One of these was excised at the top of the altered tissue, and the other was excised at the bottom of the lesion. The first sample included the epidermis, dermis and the top of the subcutaneous tissue. The second sample minimally contained the deep dermis and subcutis. The third sample was taken from the transition of the subcutaneous tissue to the underlying surface of the tuber spina scapulae.

Formalin-fixed soft tissue samples were processed via standard methods and embedded in paraffin. Formalin-fixed tissue samples containing bone tissue of the spina scapulae were decalcified with 5% HNO_3_ prior to embedding in paraffin. For histopathological examination, 2–4 μm-thick sections were prepared and stained with haematoxylin and eosin (H&E). Toluidine blue stain was further used for identification of glycosaminoglycans in neuromas.

H&E-stained sections of five samples per shoulder were examined by light microscopy using a modification of the histological criteria as described by Jensen [23] (Table 1).

Traumatic neuromas were identified according to their size in comparison with unaffected nerves.

After light microscopy examination, each shoulder was assigned to one of six stages using a simplified scheme (Table 4). Shoulder tissues were collected until a data set of 30 shoulders for each of the six stages of Score-H was confirmed through histopathological investigation.

**Table 4:** Score-H^a^ - classification

Stage	Criteria
0	No lesion
1	Lesions in epidermis and superficial dermis
2	Lesions in epidermis and entire dermis
3	Lesions in all skin layers
4	Lesions in all skin layers and underlying bone
5	Integrated epidermis with underlying granulation tissue

^a^Histopathological Score

For further analysis, the histopathological stages (Score-H) were assigned to the clinical examination findings performed before slaughter. The focus was set on the differentiation between shoulder ulcers with superficial skin layers affected and those with deeper alterations affecting all skin layers or even the bone tissue.

### Statistical evaluation

The sampling procedure was designed to compile a data set with 30 sows in each stage of the histopathological score (Score-H, see Table 4). The clinical and histopathological findings were transferred from handwritten records to a Microsoft Excel® (Version 2010, Microsoft Corporation, Redmond, Washington, USA) spreadsheet. The analysis was generated with the help of SAS®, Version 9.3 TS level 1 M2 and Enterprise Guide®, Version 7.1., SAS Institute Corporation, Cary, North Carolina, USA. Frequency tables were determined for clinical parameters according to the histopathological stages of the shoulder ulcers. Descriptive analysis was performed for possible associations of Score-H Stages 1 to 4 with clinical measurements. From this, stages 1 and 2—as well as stages 3 and 4—were combined and analysed as controls and cases by means of logistic regression techniques. A starting set of associated variables was generated for regression models with backward selection to an including level of α = 0.15. On this basis, combinations of clinical measurements characterising the histopathological stages were identified. Following the logistic regression, a prognostic curve was designed from which the likelihood of the occurrence of clinical stages of shoulder ulcers was compiled.

## Results

### Score-H – Histological characterisation

The absence of any epidermal and dermal alterations (Additional file 1) was characterising Score-H Stage 0. No traumatic neuromas were found.

An epidermis that was frequently sloughed off or replaced by a hypereosinophilic layer (Additional file 2) was characteristic for Stage 1. Rarely, bacterial colonies were found on the surface. The dermis had focal necrotic areas with single microabscesses and mild suppurative folliculitis. Mild to moderate amounts of granulation tissue, thrombosis and lymphohistiocytic infiltration could be observed in the subcutis. Traumatic neuromas were found in five of the shoulders (16.7%). The absence of the epidermis and a dermal layer that was completely necrotic or more than 50% absent was characterising Stage 2. Major parts of the dermis were replaced by granulation tissue, which was infiltrated by lymphocytes, macrophages and suppurative microabscesses. In a few samples, a mild focal necrosis and lymphohistiocytic infiltration in the subcutis could be observed. Traumatic neuromas were found in 11 shoulders (36.7%). Stages 1 and 2 frequently showed a mild proliferation of the stratum fibrosum of the periosteum. Stage 3 samples also lacked an intact epidermal layer. Granulation tissue extended from the ulcerated surface to the deep subcutaneous tissue. Marked multifocal suppurative folliculitis and larger abscesses with intralesional bacterial colonies were found in four sows. The subcutaneous tissue was frequently necrotic (Additional file 3). In two cases, the surrounding muscular tissue had focal degenerative alterations. In three cases, the dermis was nearly unaffected, although the subcutaneous tissue was largely necrotic and replaced by granulation tissue. Traumatic neuromas were found in five shoulders (16.7%). An extensive amount of granulation tissue extending to the deeper part of the subcutaneous tissue was characteristic for Stage 4. The epidermal layer was missing in all samples. The dermal and subcutaneous tissues were frequently replaced by necrotic tissue or marked granulation tissue. At the periphery of these lesions, muscle tissue with degenerative changes and granulation tissue were present. Within several samples, the different tissue layers over the bone could not be identified because of the large amount of granulation tissue. In some specimens classified as Stage 4, the bone tissue was “unprotected,” which means that the covering skin layers were no longer existent. Severe, diffuse osteolysis and osteoclastic bone resorption (Additional file 4) were found. In the bone marrow, a diffuse, marked infiltration with neutrophilic granulocytes and macrophages was found. The transition between subcutis and bone was indistinct due to the substantial amount of granulation tissue (Additional file 5). Traumatic neuromas were found in one shoulder (3.3%). Stage 5 samples lacked an epidermal layer. The dermal tissue showed a moderate, multifocal aggregation of small capillaries, which occasionally extended to the subcutis. A mild to moderate multifocal diffuse proliferation of connective tissue could be found in nearly all samples. A few samples showed multifocal microabscesses and abscesses in the deeper dermal layer or subcutis. The stratum fibrosum of the periosteum was mildly proliferated in several samples. One sample showed a prominent diffuse increase of small blood vessels in the stratum fibrosum of the periosteum. Traumatic neuromas were found in five shoulders (16.7%).

The size of traumatic neuromas was as much as 20 times the size of an unaffected nerve in the region. Other characteristics included metachromasia, due to staining of glycosaminoglycans with toluidine blue, and concentric, onion shell-like proliferation of fibrotic tissue (See Additional files 6 and 7).

### Clinical findings associated with score-H stages

For each sow, the clinical findings revealed by examination of the shoulders have been related to the individual Score-H Stage that had been determined previously (Table 5). Score-H Stage 0 shoulders showed intact skin, no scab, scar tissue, masses or powm. Hairlessness was found in 12 out of the 30 shoulders (40.0%). In Score-H Stages 1 to 4, hairlessness, scabs and reddening were frequently found. The powm was not detected in sows with lesions assigned to Score-H Stage 1, although it was identified in one sow with a stage 2 shoulder lesion. In sows with lesions assigned to stages 3 and 4, the powm was detected in 10 and 21 sows, respectively. A mass at the localisation of the shoulder lesion was also more frequently found in stage 3 and 4 shoulders, and masses with an extension of more than 4 cm were found only in these sows. In all sows assigned to Score-H Stage 5, the shoulders showed prominent scar tissue, and nearly all shoulders showed hairlessness. Sixteen of the 30 shoulders had increased reddening, and one shoulder had a fluctuating mass extending less than 4 cm in diameter.

**Table 5:** Clinical findings in shoulder lesions of Score-H^a^ Stages^b^ 0 to 5 (number of findings; % in brackets)

Clinical parameter	Stage 0		Stage 1		Stage 2		Stage 3		Stage 4		Stage 5	
Hairless	12	(40)	26	(86.7)	30	(100)	30	(100)	30	(100)	25	(83.3)
Intact	30	(100)	0	(0)	0	(0)	0	(0)	0	(0)	18	(60)
Reddening	9	(30)	21	(70)	22	(73.3)	24	(80)	24	(80)	16	(53.3)
Scratch	7	(23.3)	10	(33.3)	6	(20)	7	(23.3)	4	(13.3)	5	(16.7)
Scratches (≥ 2)	9	(30)	18	(60)	10	(33.3)	10	(33.3)	6	(20)	10	(33.3)
Scar	0	(0)	8	(26.7)	14	(46.7)	8	(26.7)	7	(23.3)	30	(100)
Powm^c^	0	(0)	0	(0)	1	(3.3)	10	(33.3)	21	(70)	0	(0)
Scab	0	(0)	30	(100)	30	(100)	30	(100)	30	(100)	12	(40)
Mass	0	(0)	5	(16.7)	8	(26.7)	19	(63.3)	28	(93.3)	1	(3.3)
Mass > 4 cm	0	(0)	0	(0)	0	(0)	5	(16.7)	16	(53.3)	0	(0)
Fluctuation of mass	0	(0)	3	(10)	2	(6.7)	5	(16.7)	3	(10)	1	(3.3)
Movable mass	0	(0)	4	(13.3)	3	(10)	8	(26.7)	3	(10)	0	(0)

^a^Histopathological Score

^b^*n* = 30 sows per Score-H Stage

^c^Proliferation of wound margin

For further analysis, Score-H Stages 1 and 2, representing lesions restricted to the superficial skin layers, and Stages 3 and 4, comprising deeper lesions affecting the subcutis or even the bone, were summarised as stage 1/2 (controls) and 3/4 (cases) (Table 6). Descriptive analysis revealed enhanced odds ratios for the detection of the variables powm, mass and more than one scratch in sows with stage 3/4 lesions compared to stage 1/2 lesions. The variables intact skin, hairlessness, scar tissue, reddening, fluctuation and movability of mass showed no association with stage 1/2 lesions, in contrast to stage 3/4 lesions (data not shown). The mean DOS of shoulder ulcers increased from a mean of 2.1 cm in stage 1/2 lesions to 4.1 cm in stage 3/4 lesions (*p* < .0001).

**Table 6:** Descriptive analysis of clinical findings potentially associated with Score-H^a^ Stages 1/2 and 3/4 (starting set for multivariable modelling)

Quantitative variable		N	Mean	Median	CV^b^	Min	5%^c^	95%^d^	Max	P^e^
DOS^f^	Stage 1/2	60	2.1	2.1	56.1	0.3	0.5	4.3	6.0	
	Stage 3/4	60	4.1	3.9	47.0	0.5	0.9	7.7	8.8	<.0001
Qualitative variables	Category	Stages 1/2			Stages 3/4			OR^g^	P^h^	Cl^i^
		N	%		n	%				
Powm^j^	no	29	48.3		59	98.3		1		
	yes	31	51.7		1	1.7		63.1	<.0001	8.2–485.1
Mass	no	13	21.7		47	78.3		1		
	yes	47	78.3		13	21.7		13.1	<.0001	5.5–31.2
> 1 scratch	no	44	73.3		32	53.3		1		
	yes	16	26.7		28	46.7		0.4	0.0244	0.2–0.9

^a^Histopathological Score

^b^Coefficient of variation

^c^5th percentile

^d^95th percentile

^e^p-value Wald’s Chi^2^-Test

^f^Diameter of scab

^g^Odds Ratio

^h^*p*-value Chi^2^-Test

^i^95% Wald’s confidence interval

^j^Proliferation of wound margins

A logistic regression model of clinical variables suspected to allow the discrimination of findings associated with shoulder ulcers Score-H Stages 3/4 from Stages 1/2 was conducted with a backwards approach to an including level of 0.15. The model shows, that strong confounding effects hamper the interpretation of univariable models. The three clinical variables ultimately identified as closely associated with these stages were DOS (OR_raw_ = 2.3, OR_adjust_ = 1.9, CI 1.3–2.7), mass (OR_raw_ = 13.1, OR_adjust_ 4.6, CI 1.6–13.0) and powm (OR_raw_ = 63.1, OR _adjust_ = 11.6, CI 1.3–104.7).

**Fig. 2:**
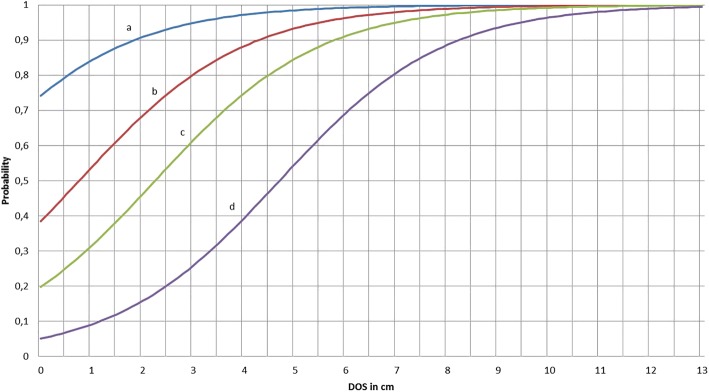
Probability curve for differentiating shoulder ulcer stages 3/4 from stages 1/2 on the basis of the logistic regression model (Scenario 1: DOS+powm+mass; Scenario 2: DOS+powm; Scenario 3: DOS+mass; Scenario 4: DOS)

Based on the adjusted Odds ratios of the results of the logistic model, prognostic curves for the probability of shoulder ulcers being stage 3/4 in contrast to 1/2 (Fig. 2) were derived, taking the different combinations of clinical measurements and DOS into account. DOS (in cm) combined with the presence of a powm and any mass showed the steepest curve progression compared to various combinations of DOS with powm or DOS with a mass or DOS alone. To secure a probability of 90% for shoulder ulcers to belong to Score-H stages 3/4, the clinical variables (especially the DOS) need to meet certain requirements. In combination with powm and mass, the DOS must be greater than or equal to 1.9 cm (3.1 cm). The DOS combined with powm needs to be greater than or equal to 4.3 cm (5.5 cm). In combination with the presence of a mass, the DOS needs to be greater than or equal to 5.8 cm (7.0 cm). Shoulder ulcers solely showing DOS need to be greater than or equal to 8.3 cm (9.5 cm).

## Discussion

Under conventional farming conditions, shoulder ulcers are frequently occurring lesions in sows following farrowing. The herd prevalence can vary from approximately 10% to nearly 40% [5, 6]. Shoulder ulcers are lesions that are often underestimated in terms of pain. Pain in shoulder ulcers is likely caused by local inflammatory reactions, as well as by traumatic neuromas [4, 24]. Clinical symptoms such as rubbing behaviour are indicative of pain reactions related to shoulder ulcers [4, 17]. The presence of shoulder ulcers in sows without pain-relieving therapy is a significant welfare problem [2, 4, 23]. From findings in human beings, it can be extrapolated that an ulcer affecting the deeper skin layers is more painful compared to ulcers restricted to the superficial layers [1]. In our study, traumatic neuromas were detected in 27% of Score-H Stage 1 and 2 and 10% of Stage 3 and 4 shoulder ulcers. The lower detection rate for Stage 3 and 4 shoulder ulcers might be influenced by the standardised size of the sample. Therefore, in shoulder ulcers with a lesser degree of granulation tissue, the samples included more bordering tissue compared to ulcers with a larger degree of granulation tissue.

The severity of shoulder ulcers in sows is commonly classified with the help of clinical or histopathologic scores. These scores are based on the depth of the lesion, defined based on the affected skin layers and bone tissue. Ulcers restricted to the epidermis and dermis can be assessed as insubstantial animal welfare-related lesions, and ulcers involving the subcutis or even the bone tissue can be evaluated as substantial animal welfare-related lesions. The discrimination between substantial and insubstantial animal welfare-related ulcers refers to the scoring and evaluation of ulcers in human beings [11]. The histopathological scoring of shoulder ulcers in sows has been described in various publications [23, 25] and is comparatively easy to practice. In contrast, differentiation can be difficult clinically, as the depth of the lesion is hard to determine in a clinical examination due to the thin skin covering the bony prominence of the tuber spina scapulae. Wound secretions and the presence of granulation tissue are aggravating factors that make exploration via probing even more challenging. The question therefore arises regarding which clinical measurements are associated with the histopathological stages (the reference standard measurement) and how these stages can be differentiated. To achieve a clearer clinical contrast, the Score-H Stages 1 and 2 and Stages 3 and 4 have been summarised. The clinical parameters best fitting with the respective stages of the histopathological score were allocated to a newly contrived clinical score (Score-C). This novel score comprises the stages I to IV. Stages II and III represent the dividing line between insubstantial and substantial animal welfare-related shoulder ulcers. The latter can be predicted with the help of a prognostic curve showing the likelihood of identifying a Stage III shoulder ulcer when certain clinical parameters or combinations of parameters are present (Fig. 2).

Stage I shoulders have intact skin without any alterations. This definition is consistent with the first stage of scores previously reported [22, 23, 26]. Several authors also include acute fighting wounds in this first stage [5] or even ulcerative lesions with a diameter less than two centimetres (Danish Score, see Table 2). However, the results of the present study show that even lesions less than 2 cm in diameter could extend to deeper skin layers and should not be assigned to the stage comprising shoulders with intact skin. Shoulders without open wounds but with scar tissue have also not been assigned to Stage I, as suggested by Zurbrigg [26], but to a separate Stage IV. Scar tissue in the skin over the tuber spina scapulae is indicative of a former shoulder ulcer covered by healed skin [2, 27]. Healed shoulder ulcers with scar tissue have a higher risk of developing a relapsed acute ulcer during subsequent farrowing [27], which justifies categorisation in Stage IV. Shoulder ulcers restricted to superficial layers of the epidermis are grouped in Stage II. The probability of detecting such ulcerations is 90% with a DOS less than 1.2 cm. The clinical scores previously published associate mild lesions, comparable to Stage II, with scab diameters up to 2.3 cm [26] or 2–5 cm (Danish Score), but these scores have not been compared to histopathological findings and cannot be associated with the affected skin layers.

Stage III shoulder ulcers, which extend to the deeper tissue layers, the subcutis and occasionally also to the bone tissue, can be identified with the help of the diameter of the scab and with the presence or absence of masses and powm. A shoulder ulcer showing powm, a mass and a DOS of 1.9 cm or more can be categorised as Stage III with a probability of 90%. If an ulcer shows solely clinical signs of powm, the DOS needs to be at least 4.3 cm to be correctly classified as Stage III with a probability of 90%. Shoulder ulcers showing only a mass need a diameter of at least 5.8 cm to be assigned with a 90% probability to Stage III. Lesions without any powm or mass require a DOS of at least 8.3 cm to be categorised as Stage III with a 90% probability.

The above-described clinical parameters or combination of clinical parameters representing Stage III are associated with histopathological lesions involving all skin layers (and also the underlying bone tissue in some cases). The same clinical findings have been categorised as less severe (Stage 1) in the Danish Score or as Stage 3 of the four-stage score published by Zurbrigg [26]. The differences in the clinical scores previously reported (Table 2) and the newly developed score are most likely the result of the comparison to the histopathological findings, which have been set as the “reference standard”. Furthermore, the informative value of combinations of clinical parameters has not been the focus of former studies (Table 2). The results of this study show that certain clinical findings or combinations of findings with the DOS allow classification of Stage III shoulder ulcers with a probability of 90%. Clinical examination scores usually have a lower sensitivity and specificity [28–30] compared to commonly used laboratory tests such as ELISA or PCR. Therefore, a 90% probability for classification as Stage III can be considered sufficient.

## Conclusions

This study focused on the development of a score for use in the clinical evaluation of shoulder ulcers in sows. Various clinical findings were characterised for their association with histopathological findings (the “reference standard” analytical methodology). The presence or absence of proliferation of the wound margin, mass and the diameter of the scab were the findings fitting best to the stages of the histopathological score. The clinical score developed enables farmers, veterinary practitioners and public veterinarians to categorise shoulder ulcers in sows with the help of a four-stage clinical score and a prognostic curve. This novel score particularly helps in the discrimination of shoulder ulcers restricted to the superficial skin layers (insubstantial animal welfare-related lesions) from shoulder ulcers involving all skin layers and sometimes even the underlying bone, which must be assessed as substantial animal welfare-related lesions.

## Additional files

Additional file 1: Intact epidermis (E) and intact dermis (D) from a shoulder ulcer Stage 0, H&E. (TIF 14706 kb)

Additional file 2: Replacement of epidermis by a hypereosinophilic layer (EL) and intact dermis (D) of a shoulder ulcer Stage 1; Hair follicle (HF), Blood vessel (B), H&E. (TIF 14603 kb)

Additional file 3: Necrotic area at the transition (TS) between the dermis (D) and subcutis (S) infiltrated with neutrophilic granulocytes and macrophages; Pyknotic cell fragments (arrows), H&E. (TIF 14716 kb)

Additional file 4: Osteolytic process within an “unprotected bone”; Fragment of bone tissue (B) surrounded by osteoclasts (arrows) and restorative changes from granulation tissue (G), H&E. (TIF 18557 kb)

Additional file 5: Section of bone tissue of the tuber spina scapulae (B) surrounded by granulation tissue (G) and prominent areas of necrosis (*), H&E. (TIF 18679 kb)

Additional file 6: Cross-section of a traumatic neuroma in a Stage 1 shoulder ulcer with concentric, onion shell-like proliferation of fibrous tissue, H&E. (TIF 18576 kb)

Additional file 7: Traumatic neuroma (same as in Additional file 6) with metachromatically stained glycosaminoglycans (arrows), TB. (TIF 18728 kb)

## Abbreviations

BCS: Body condition score
DOS: Diameter of scab
H&E: Haematoxylin and Eosin
HNO3: Nitric acid
IS: Intact skin
POWM: Proliferation of wound margins
Score-C: Clinical score
Score-H: Histopathological score
