# Supplementary figures and images for: Scoring shoulder ulcers in breeding sows – is a distinction between substantial and insubstantial animal welfare-related lesions possible on clinical examination?
Source: Porcine Health Manag. 2019 Jan 22;5:3. doi: 10.1186/s40813-018-0108-3 (PMC6341639; doi:10.1186/s40813-018-0108-3)

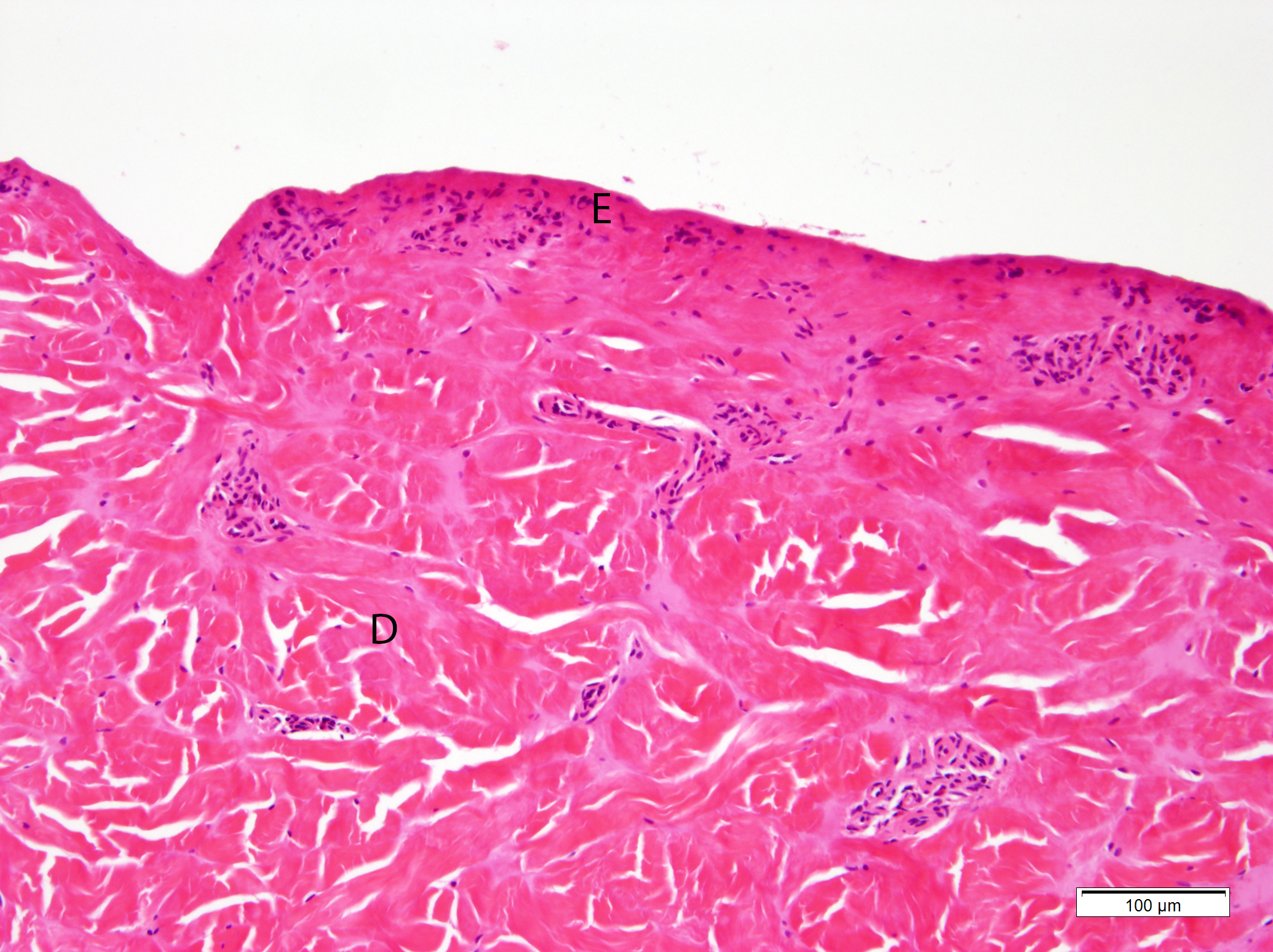

Supplement: Supplementary file 1 — Intact epidermis (E) and intact dermis (D) from a shoulder ulcer Stage 0, H&E. (TIF 14706 kb) [file 40813_2018_108_MOESM1_ESM.tif]

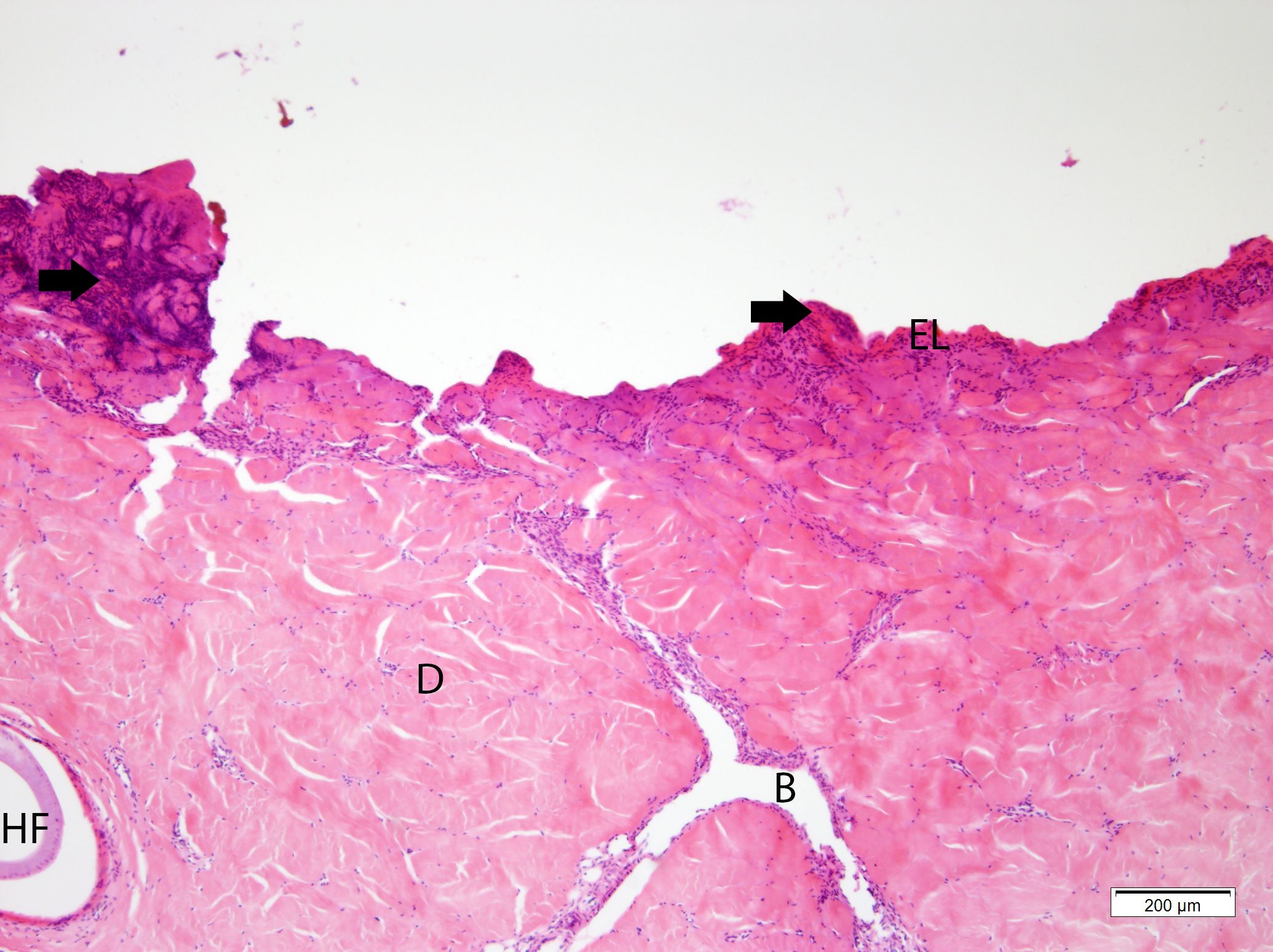

Supplement: Supplementary file 2 — Replacement of epidermis by a hypereosinophilic layer (EL) and intact dermis (D) of a shoulder ulcer Stage 1; Hair follicle (HF), Blood vessel (B), H&E. (TIF 14603 kb) [file 40813_2018_108_MOESM2_ESM.tif]

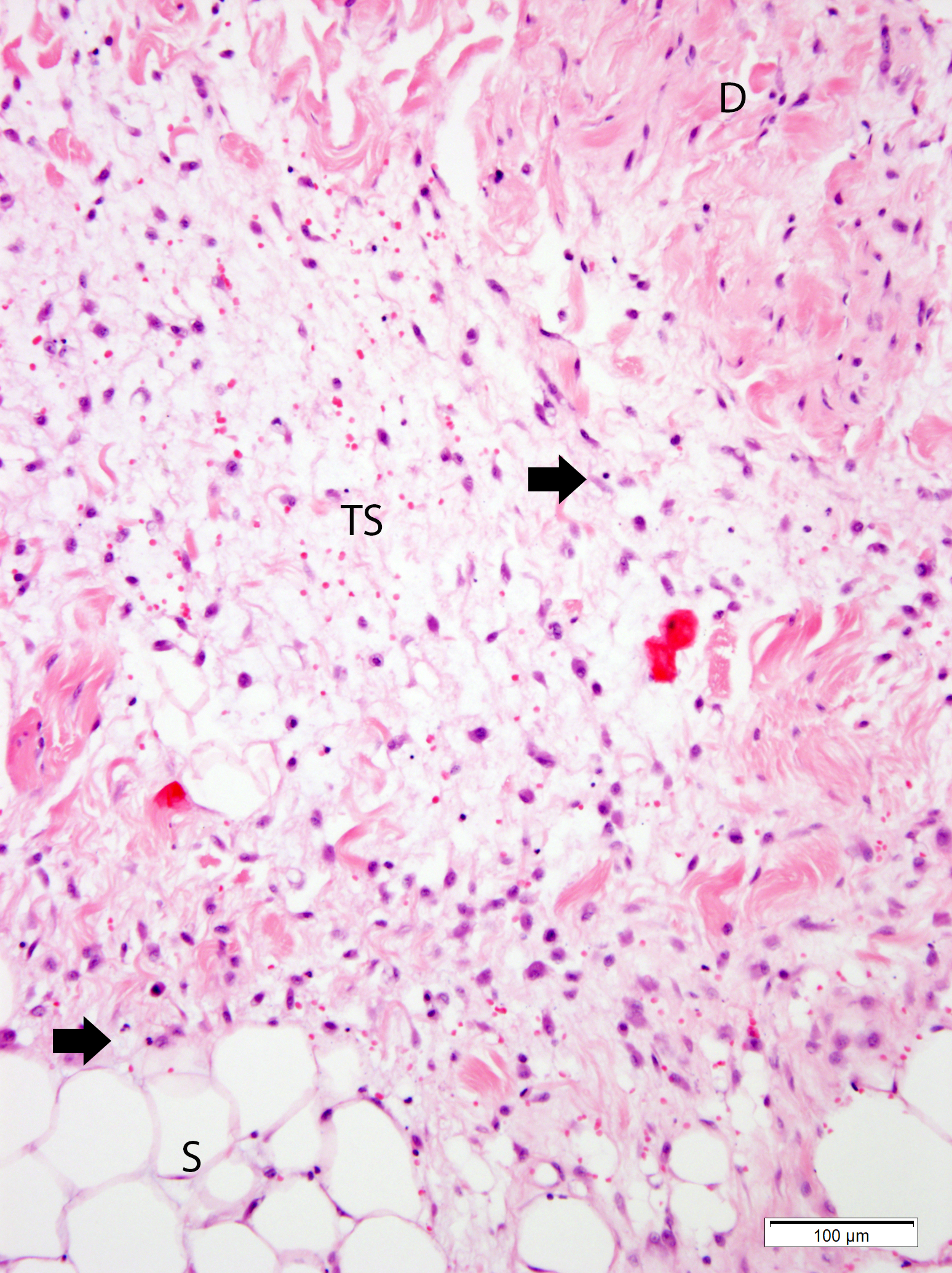

Supplement: Supplementary file 3 — Necrotic area at the transition (TS) between the dermis (D) and subcutis (S) infiltrated with neutrophilic granulocytes and macrophages; Pyknotic cell fragments (arrows), H&E. (TIF 14716 kb) [file 40813_2018_108_MOESM3_ESM.tif]

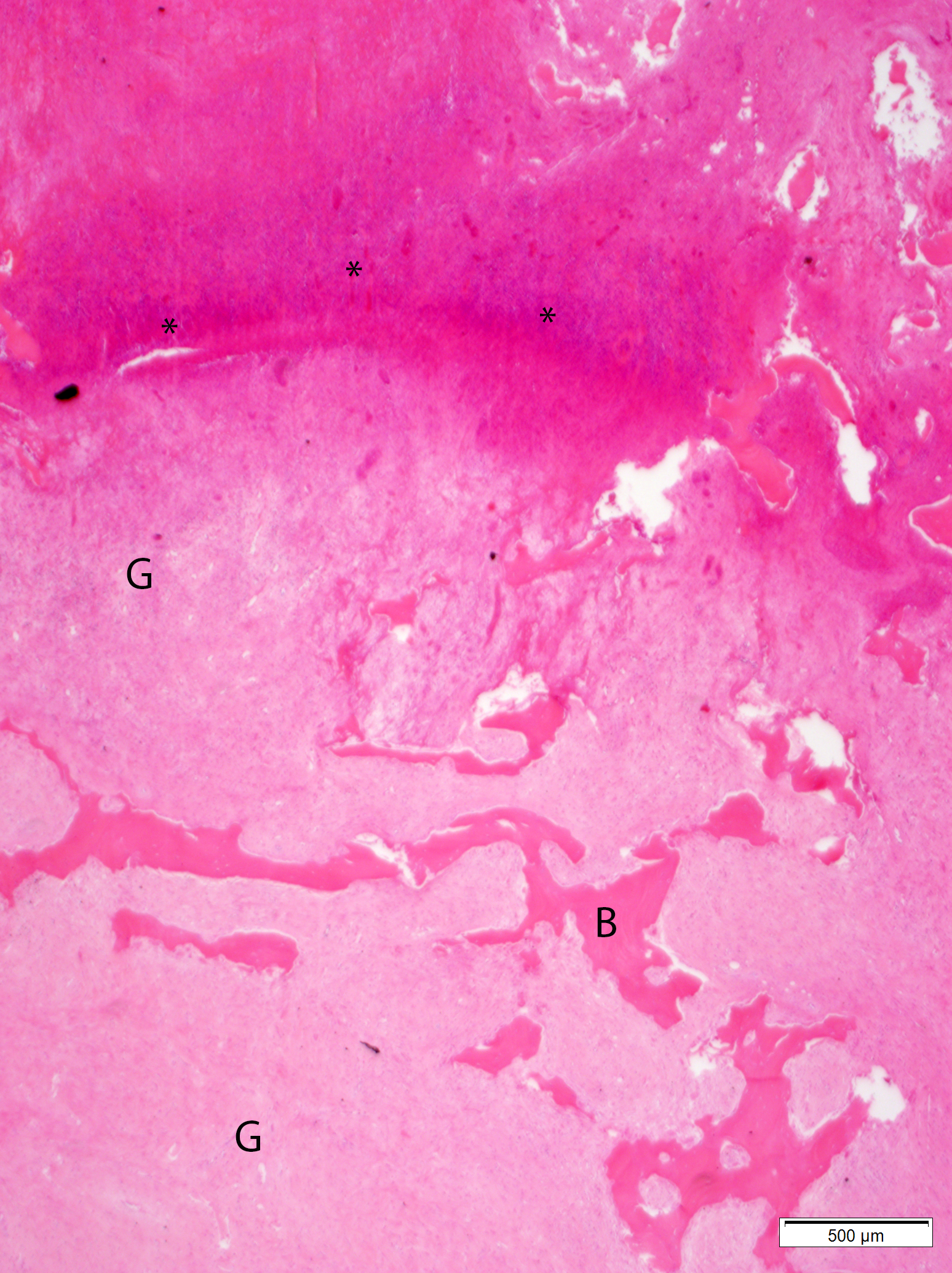

Supplement: Supplementary file 5 — Section of bone tissue of the tuber spina scapulae (B) surrounded by granulation tissue (G) and prominent areas of necrosis (*), H&E. (TIF 18679 kb) [file 40813_2018_108_MOESM5_ESM.tif]

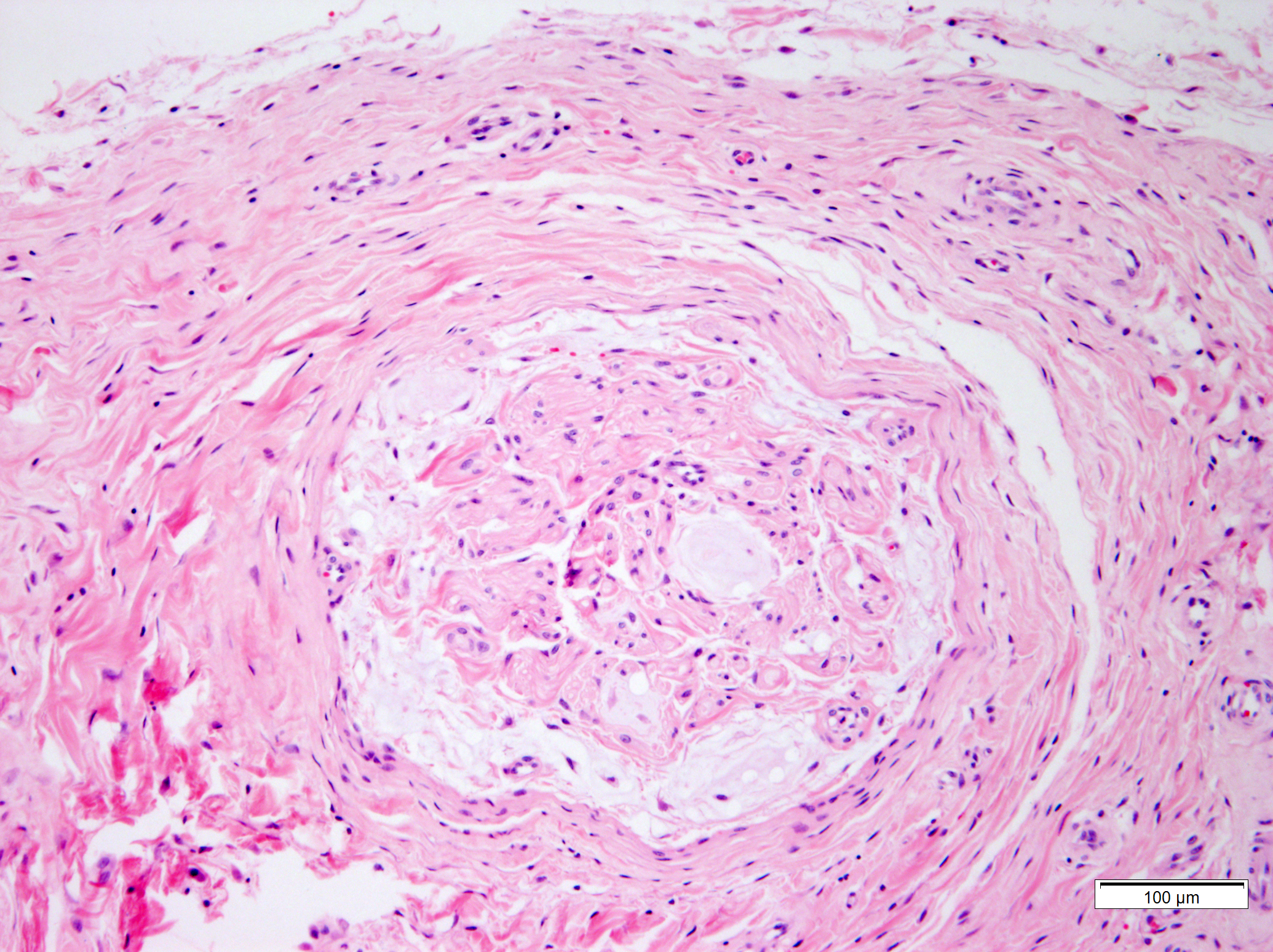

Supplement: Supplementary file 6 — Cross-section of a traumatic neuroma in a Stage 1 shoulder ulcer with concentric, onion shell-like proliferation of fibrous tissue, H&E. (TIF 18576 kb) [file 40813_2018_108_MOESM6_ESM.tif]

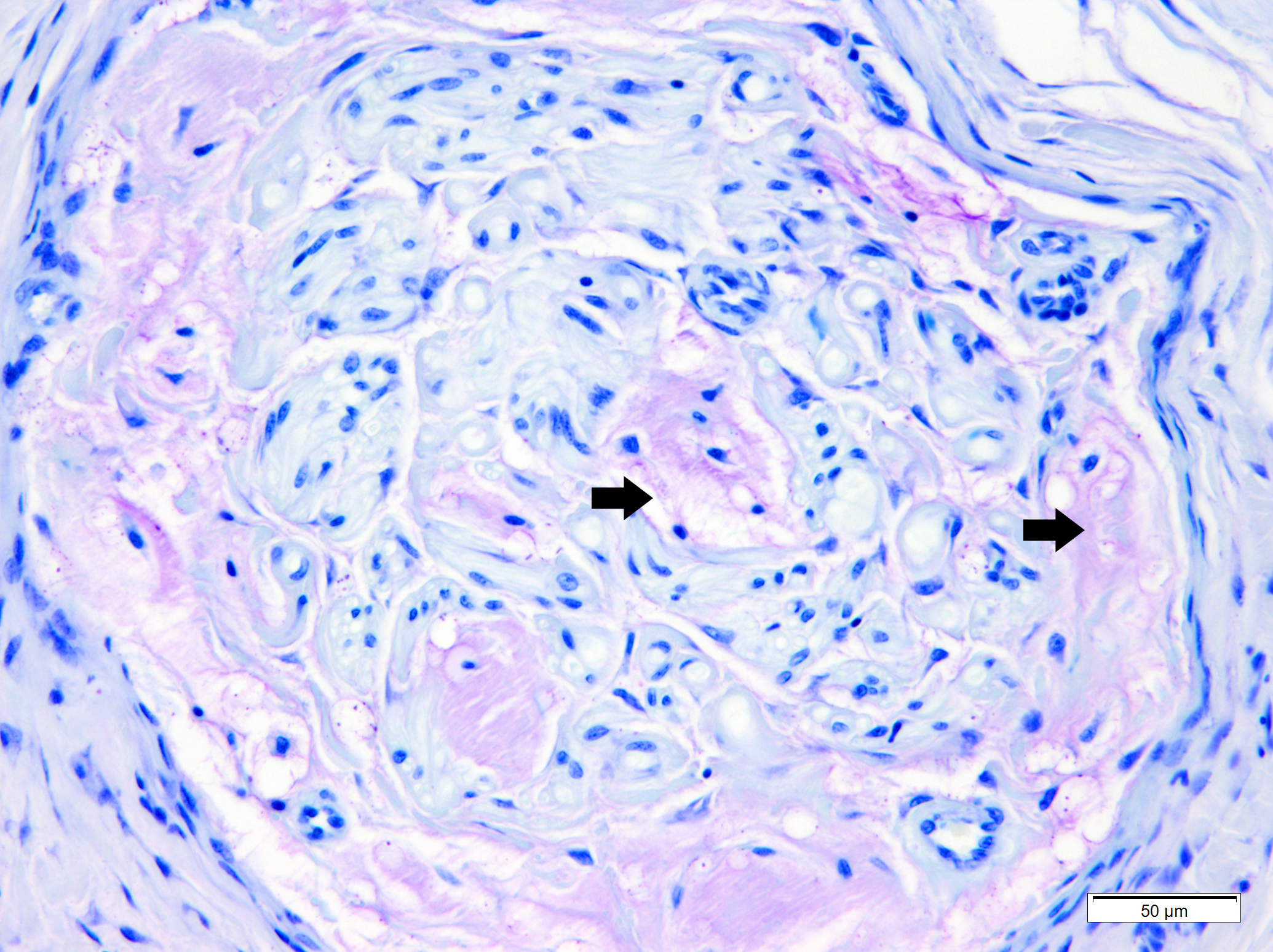

Supplement: Supplementary file 7 — Traumatic neuroma (same as in Additional file 6) with metachromatically stained glycosaminoglycans (arrows), TB. (TIF 18728 kb) [file 40813_2018_108_MOESM7_ESM.tif]
